# Measurement of CYP1A2 and CYP3A4 activity by a simplified Geneva cocktail approach in a cohort of free-living individuals: a pilot study

**DOI:** 10.3389/fphar.2024.1232595

**Published:** 2024-02-02

**Authors:** Constance A. Sobsey, Noor Mady, Vincent R. Richard, Andre LeBlanc, Thomas Zakharov, Christoph H. Borchers, R. Thomas Jagoe

**Affiliations:** ^1^ Segal Cancer Proteomics Centre, Lady Davis Institute for Medical Research, Jewish General Hospital, McGill University, Montreal, QC, Canada; ^2^ Division of Experimental Medicine, Faculty of Medicine, McGill University, Montreal, QC, Canada; ^3^ Peter Brojde Lung Cancer Centre, Jewish General Hospital, Montreal, QC, Canada; ^4^ Gerald Bronfman Department of Oncology, Jewish General Hospital, McGill University, Montreal, QC, Canada; ^5^ Department of Medicine, Jewish General Hospital, Montreal, QC, Canada

**Keywords:** cytochrome P450, precision dosing, personalized medicine, precision oncology, Geneva cocktail, drug-drug interaction, adverse drug reaction, pharmacokinetics

## Abstract

**Introduction:** The cytochrome P450 enzyme subfamilies, including CYP3A4 and CYP1A2, have a major role in metabolism of a range of drugs including several anti-cancer treatments. Many factors including environmental exposures, diet, diseaserelated systemic inflammation and certain genetic polymorphisms can impact the activity level of these enzymes. As a result, the net activity of each enzyme subfamily can vary widely between individuals and in the same individual over time. This variability has potential major implications for treatment efficacy and risk of drug toxicity, but currently no assays are available for routine use to guide clinical decision-making.

**Methods:** To address this, a mass spectrometry-based method to measure activities of CYP3A4, CYP1A2 was adapted and tested in free-living participants. The assay results were compared with the predicted activity of each enzyme, based on a self-report tool capturing diet, medication, chronic disease state, and tobacco usage. In addition, a feasibility test was performed using a low-volume dried blood spots (DBS) on two different filter-paper supports, to determine if the same assay could be deployed without the need for repeated standard blood tests.

**Results:** The results confirmed the methodology is safe and feasible to perform in free-living participants using midazolam and caffeine as test substrates for CYP3A4 and CYP1A2 respectively. Furthermore, though similar methods were previously shown to be compatible with the DBS format, the assay can also be performed successfully while incorporating glucuronidase treatment into the DBS approach. The measured CYP3A4 activity score varied 2.6-fold across participants and correlated with predicted activity score obtained with the self-report tool. The measured CYP1A2 activity varied 3.5-fold between participants but no correlation with predicted activity from the self-report tool was found.

**Discussion:** The results confirm the wide variation in CYP activity between individuals and the important role of diet and other exposures in determining CYP3A4 activity. This methodology shows great potential and future cross-sectional and longitudinal studies using DBS are warranted to determine how best to use the assay results to guide drug treatments.

## 1 Introduction

The concept of “precision dosing” of cancer chemotherapy includes the adjustment of drug doses and intervals to maximize cancer treatment efficacy for each patient, while minimizing toxic effects of anti-cancer agents (Darwich et al., 2021). Adjusting drug dosing using drug monitoring (TDM) methods that measure circulating levels of individual cancer drugs is one obvious solution, but this is not yet possible for most drugs as it requires rigorous assay development and validation for each and every new drug (Darwich et al., 2021). However, other approaches including more comprehensive patient characterization have potential to greatly enhance routine care and guide cancer treatment dosing (Darwich et al., 2021). One example of the latter approach is to determine cytochrome P450 enzyme activity in individual patients. Most cancer drugs are metabolized by one or more of the cytochrome P450 enyzmes (CYPs), principally those located in the endoplasmic reticulum of liver cells, though some drug-metabolizing CYPs are found in the mitochondrial membrane (Fujita, 2006; Zhao et al., 2021). Importantly, activity of these enzymes varies widely between individuals and, even in the same individual, CYP activities can change over time depending on a range of other factors. These inter-individual differences in CYP activity have been linked to altered treatment effectiveness and overall survival for both conventional chemotherapeutics and new targeted agents such as tyrosine kinase inhibitors (Michael and Doherty, 2005; Petros et al., 2005; Daali et al., 2021). To date, clinical testing of CYP activity prior to, or during cancer treatment, is not available and there is a pressing need to address this critical knowledge gap in advancing individualized cancer drug dosing.

Research in precision dosing with respect to CYP activity initially focused on a pharmacogenetic approach. By identifying the presence of known CYP gene polymorphisms with different activities, the metabolism of drugs which were substrates for a given CYP subfamily was predicted (Rochat, 2005; Fujita, 2006; Shastry, 2006). This approach showed some promise as genetic polymorphisms are associated with treatment response in some cases (Seredina et al., 2012). However, genetic sampling presents challenges for clinical implementation, particularly since many genetic variants of interest are present at low frequency, and results from one cohort often cannot be generalized to another (Cronin-Fenton and Damkier, 2018; Bienfait et al., 2022; Sukri et al., 2022). Over 30 different CYP3A4 variants have been described, which can lead to modest increased, normal or decreased activity. However, none of these variants is common and the combined frequency of all variants is low (<6%) in the population (Zhou et al., 2017). By contrast, genetic variants of CYP1A2 vary more widely in their effect on enzyme activity and inducibility. Most are only present at low frequency in the general population. The exception is the CYP1A2*1F variant, which causes CYP1A2 to be highly inducible and is present at much higher frequencies (i.e. 55%–73% of the population depending on ethnicity) (Solus et al., 2004; Gaedigk, 2023). Polycyclic aromatic hydrocarbons present in tobacco smoke induce CYP1A2 activity (O’Malley et al., 2014) and this is particularly marked in smokers expressing CYP1A2*1F variant (Sachse et al., 1999).

Precision-dosing based solely on CYP genetic polymorphism characterization is also flawed as it does not account for the large inter- and intra-individual differences in activity of CYP enzymes due to induction or inhibition by environmental or disease factors. These include drugs, foods and other dietary factors, natural health products and other exposures such as smoking, all of which are also well-known environmental factors that can play an important role in determining treatment response (Anderson and Kappas, 1991; Delgoda and Westlake, 2004; Samer et al., 2013; McGraw et al., 2018; Wishart et al., 2018). In addition, many patient characteristics, including age, nutritional status, and disease progression and inflammation, also vary over time and can significantly alter the level of CYP activity present over the course of treatment (Kinirons and O'Mahony, 2004; Scripture et al., 2005; Harvey and Morgan, 2014). Over-expression of specific CYPs has been observed in tumours and progressive CYP over-activation is now implicated as a mechanism of resistance to cancer treatments (Michael and Doherty, 2005; Rochat, 2005; van Eijk et al., 2019). In fact, CYPs themselves are now under consideration as an independent target for therapeutic intervention (Michael and Doherty, 2005; Bruno and Njar, 2007; Zhao et al., 2019; Liu et al., 2021; Singh et al., 2023).

Suitable clinically practical methods are needed to measure CYP activity rapidly and repeatedly during cancer treatment. Several “cocktail” approaches have been developed to simultaneously measure the activity of multiple drug-metabolizing CYPs and transporters (Bosilkovska et al., 2014a; Bosilkovska et al., 2014b; Derungs et al., 2016). The cocktail approach involves plasma sampling and measuring the circulating concentrations of selected substrates for target CYPs and their respective primary metabolite at one or more specified timepoint(s) after administration of the cocktail. The ratio of the substrate to its metabolite provides a surrogate measure of the activity of the associated CYPs and transporters (Tornio et al., 2019).

The published methods for CYP activity assessment are frequently time-consuming for participants and require multiple blood samples, making them difficult to use in larger clinical populations. To address this, we used a simplified version of the Geneva cocktail (Bosilkovska et al., 2014b) to assess the activity of CYP1A2 and CYP3A4 in free-living volunteers. The research project was initially conceived in the Peter Brojde Lung Cancer Centre. CYP1A2 and especially CYP3A4 were selected for their relevance to metabolism of drugs used in treatment of lung cancer (Engels et al., 2004; van Erp et al., 2009). The test substrates for these CYPs (caffeine and midazolam respectively) were chosen as they are both well tolerated at the doses used and reach peak concentration at 60 min, which is a reasonable assay time interval for clinical use.

In contrast to prior studies using only healthy younger controls, we recruited participants with a broader age range, living in the community with or without chronic, stable, non-malignant disease and without restrictions on usual diet or medication use. The results in these free-living participants were used to assess the clinical feasibility of this modified protocol prior to testing in patients with lung cancer. Additionally, we documented the use of known inducers and inhibitors of each tested CYP using a structured questionnaire and semi-quantitative scoring system and correlated the results with activity of respective CYPs. To further facilitate the application of this approach for detecting differences in CYP activity in clinical practice, the assay method underwent further testing for use with dried blood spot (DBS) rather than serum samples.

## 2 Methods

### 2.1 Patients and sample collection

The study protocol was approved by the Ethics board at the Jewish General Hospital in Montreal, Canada (Study number: CODIM-MBM-16-235). Ten (10) free-living adult participants with no acute illness were recruited to the study. Participants with known allergies or intolerance to either caffeine or midazolam, with active cancer or on cancer treatment, suffering an acute illness in the prior 2 weeks, or who started a new prescription medication in the prior 6 weeks were excluded. Participants were instructed to fast overnight for a minimum of 8 h prior to testing, including abstinence from any drink other than water. Upon arrival at the test centre, a single venous blood sample was taken from the arm at “baseline” immediately prior to ingestion of a fixed oral dose of 100 mg caffeine tablet (Adrem Brands Inc.) and 2 mg midazolam (1 mg/mL liquid, Sandoz Canada Inc., mixed with ∼50 mL apple juice) to act as probes for CYP1A2 and CYP3A4 respectively. A second venous blood sample was taken 60 min following caffeine and midazolam dosing. Collected blood samples were allowed to clot at room temperature for no more than 1 h before centrifuging at 1,500 × g for 10 min to obtain serum, which was frozen in cryovials and stored at −80°C until analysis.

### 2.2 Clinical data, dietary questionnaire and scoring

Participants’ height, weight, biological sex, and age were recorded during the visit. Participants were further asked to complete a targeted dietary and medical history questionnaire (see Supplementary File S1). Items in the dietary questionnaire were identified as inducers or inhibitors of CYP3A4 or CYP1A2 based on known effects on these enzymes (Supplementary Table S2.1). Medications identified by participants were also assessed for likely inducing or inhibiting effects (Supplementary Table S2.2) (Feierman et al., 2003; Harris et al., 2003; Flockhart, 2007; Foti and Wahlstrom, 2008; Samer et al., 2013). Each predicted inducer (↑) or inhibitor (↓) of a given enzyme was assigned a “strength” (STR, low = 1, high = 2) based on the reported effects on enzyme activity. To create a weighted inducing or inhibiting score for each inventory item, the strength of a given effect was multiplied by a score assigned for “frequency of exposure” (FoE, low = 0, medium = 1, high = 2, see Table 1).

**TABLE 1:** Frequency scoring of dietary, herbal and medical substance use.

Frequency reported on questionnaire	Associated exposure level	Score assigned
“*never or rarely*” or “*1–3 times per month*”	Low	0
“*1–3 times per week*” or “*4–6 times per week*”	Medium	1
“*1–2 times per day*” or “*3 or more times per day*”	High	2

As shown in Equation 1, for each CYP enzyme, the sum of the weighted scores for all inhibiting inventory items was deducted from the sum of the weighted scores for all inducing inventory items to generate an aggregate score reflecting total predicted activity. For example, for CYP3A4, if the subject consumed one strongly inducing drug (score 2), daily (score 2) and one mildly inducing food item (score 1), four times a week (score 1) as well as daily consumption (score 2) of a mildly inhibiting food (score 1), their net Diet and Medication Questionnaire score was: DiMQu_3A4_ = ((2 × 2)+(1 × 1))–(2 × 1) = 3.

Equation 1 Example calculation of DiMQu score for a given patient for CYP3A4

Item X′s weighted inducing score for CYP3A4 WIS3A4↑x=Strength of Effect (STR3A4↑x) x Patient‐Reported Frequency of Exposure FoE↑x

Item Y′s weighted inhibiting score for CYP3A4 WIS3A4↓y=Strength of Effect (STR3A4↓y) x Reported Frequency of Exposure FoE↓y

CYP3A4 total inducing score ↑TIS3A4=∑WIS3A4↑a+WIS3A4↑b…+WIS3A4↑z

CYP3A4 total inhibiting score ↓TIS3A4=∑WIS3A4↓a+WIS3A4↓b…+WIS3A4↓z

Patient–specific DiMQu3A4 score=↑TIS3A4−↓TIS3A4

### 2.3 Analysis of CYP1A2 and CYP3A4 activity

Participant blood sample analysis was performed as previously described using liquid chromatography tandem mass spectrometry (LC-MS/MS) to quantify caffeine and paraxanthine as markers of CYP1A2 and midazolam and OH-midazolam as metabolic markers of CYP3A4 activity (Bosilkovska et al., 2014a). Briefly, we obtained high-purity commercially available standards (Sigma) for all four analytes [Midazolam, 1-OH-Midazolam, Caffeine, Paraxanthine] together with their corresponding isotope-labeled internal standards [Midazolam-D4 maleate, α-Hydroxymidazolam-D4, Caffeine-(trimethyl-d9) and 1,7-Dimethylxanthine-(dimethyl-d6)]**.** Standards were dissolved in 60% methanol and added to pooled charcoal-stripped human serum from healthy donors (BioIVT, Westbury, NY) to establish response curves to enable quantitation from participant serum.

Participant serum samples were prepared by thawing on ice. Internal standards of a known concentration were added to 50 μL aliquots of each sample, after which the samples were pre-treated with 500 units of β-glucuronidase (Sigma, Cat# G7017) for 16-h incubation at 37°C to ensure full recovery of the glucuronidated metabolic intermediate of OH-midazolam, as required for a more accurate metabolic ratio (Derungs et al., 2016). Proteins were then precipitated from the sample with 150 µL methanol containing stable isotope labeled internal standards (SIS) and centrifugation. The supernatants were diluted 1:1 in LC-MS grade H2O before injection. Samples were then analyzed via 2 μL injections (for midazolam/OH-midazolam) or 18 μL injections (caffeine/paraxanthine) on an Agilent 1,290 Infinity liquid chromatography system fitted with a Zorbax Eclipse plus column (RRHD C18, 2.1 × 15 mm, 1.8 um) at 50°C with a flow rate of 0.6 mL/min. The LC system was coupled in line to an Agilent 6495B triple quadrupole mass spectrometer (Agilent Technologies, Santa Clara, CA). Gradient-elution was performed over a 5-min gradient from 2% to 100% acetonitrile (Mobile phase A: H2O, 0.1% FA, mobile phase B: ACN, 0.1% FA). MS settings and MRM transitions are shown in Supplementary File S3. The same analytical method was used for both validation and sample analysis. Blanks, double-blanks, and QC samples were prepared and injected in parallel with the patient samples. Data from the multiple reaction monitoring (MRM) assay for each analyte was processed in Skyline software (https://skyline.ms/). The metabolic ratio (MR) for each pair of metabolites was derived by dividing the measured concentration of Hydroxymidazolam by Midazolam and the concentration of Paraxanthine by Caffeine for each sample.

### 2.4 Dried blood spot analysis

Dried blood spot (DBS) analysis was performed to assess the feasibility of using low-volume DBS samples for this protocol. Both Whatman 903 and HemaSpot HF devices were tested to compare the assay’s performance on these two substrates. Whatman 903 is one of the most common DBS cards currently in use and is typically sampled with a standardized punch. The HemaSpot HF device is designed to offer simplified sampling of pre-cut “petals,” reduce hematocrit effects on sample distribution, and irreversibly seal after collection for secure shipping and storage. In order to test the suitability of the protocol for application to DBS, “test samples” were prepared in two different ways: 1) for initial assessment of the linear range, calibration curve samples were prepared by spiking charcoal-stripped pooled whole blood from anonymized donors with varying concentrations of the analytical standards for caffeine, paraxanthine, midazolam and OH-midazolam; and 2) for further analyses, pooled participant serum samples from the 1 h timepoint were diluted 1:1 with pooled red blood cells from anonymized donors. These mixtures, which all included caffeine, paraxanthine, midazolam, and OH-midazolam, were volumetrically spotted (50 μL per spot) onto Whatman 903 DBS cards or Hemaspot HF collection devices (SpotOn Sciences, Austin, TX). For extraction, entire DBS spots were excised from Whatman 903 cards, or two petals were used for Hemaspot HF. For deconjugation of hydroxymidazolam glucuronide, two workflows were tested using either: (i) on-spot deconjugation, or (ii) an initial aqueous extraction followed by β-glucuronidase treatment. Briefly, for on-spot deconjugation, 500 units of β-glucuronidase in water (50 µL) was added directly to the DBS specimens and incubated for 16 h at 37°C. Metabolites were then extracted from the DBS by addition of 200 µL of 80% methanol containing SIS and shaking with an Eppendorf Thermomixer at 2,000 rpm at 37°C for 10 min. Samples were briefly centrifuged (2,000 × g for 5 min), and then the supernatant was processed using the protocol described for serum samples above. For sequential extraction and deconjugation, DBS specimens were first extracted with 500 µL of water for 10 min using an Eppendorf Thermomixer (2,000 rpm, 37°C), vacuum concentrated, and rehydrated in 50 µL of water prior to protein precipitation and processing as described above. The same MRM-MS assay was used to quantify analytes after extraction from DBS samples.

### 2.5 Statistical analysis

Descriptive statistics were applied to patient characteristics. R statistical programming language (version 3.5.3) was used to create scatterplots of DiMQu_3A4_ scores *versus* Hydroxymidazolam:Midazolam metabolic ratios (MRs) and DiMQu_1A2_ scores *versus* Paraxanthine:Caffeine MRs. Correlations between the variables were assessed using Spearman’s rank-order correlation. Correlations were reported as *r*_*s*_ values with a two-tailed *p*-value for determining statistical significance.

## 3 Results

### 3.1 Participant characteristics

A total of 10 participants (mean age 38 ± 11.8; *n* = 5 males) took part in the study. Most were either staff or graduate students at the Jewish General Hospital, and all were non-smokers. As shown in Table 2, 3 individuals reported chronic illnesses, 4 reported the use of natural health products (NHPs) including melatonin, vitamins, and herbal supplements, and 6 reported medication use (further details in Supplementary Table S2.2). No participants reported any adverse effects, specifically no drowsiness with this oral dose of midazolam.

**TABLE 2:** Participant characteristics.

	*n* = 10	—
Age (yr)	mean 38 (±11.8)	Range: 26–60
BMI (kg/m^2^)	mean 23.2 (±1.7)	Range: 21–26
Male	5	—
Chronic illness	3	—
Smokers	0	—
Natural health product use	4	—
Current medication use	6	—

**TABLE 3:** CYP3A4 Metabolic Ratio. No reported value (−) indicates that the measured value was below the LLOQ. Total OH-midazolam is the measured concentration after glucuronidase pre-treatment of the sample. The metabolic ratio is calculated as total OH-Midazolam at 1 h divided by Midazolam at 1 h.

		Midazolam (ng/mL)		OH-midazolam (ng/mL)			Metabolic ratio
					Free	Total	
		0 h	1 h	0 h	1 h	1 h	1 h
Participant							
**1**		-	7.35	-	7.86	47.9	6.52
**2**		-	7.19	-	4.70	61.5	8.56
**3**		-	10.9	-	8.95	76.0	6.96
**4**		-	7.13	-	6.57	71.0	9.97
**5**		-	6.69	-	4.15	47.1	7.04
**6**		-	4.46	-	4.60	59.7	13.4
**7**		-	7.29	-	5.93	49.4	6.78
**8**		-	8.97	-	9.01	53.3	5.94
**9**		-	4.97	-	3.97	25.3	5.09
**10**		-	7.42	-	6.85	48.4	6.52

### 3.2 Predicted CYP activity score (DiMQu questionnaire)

**FIGURE 1:**
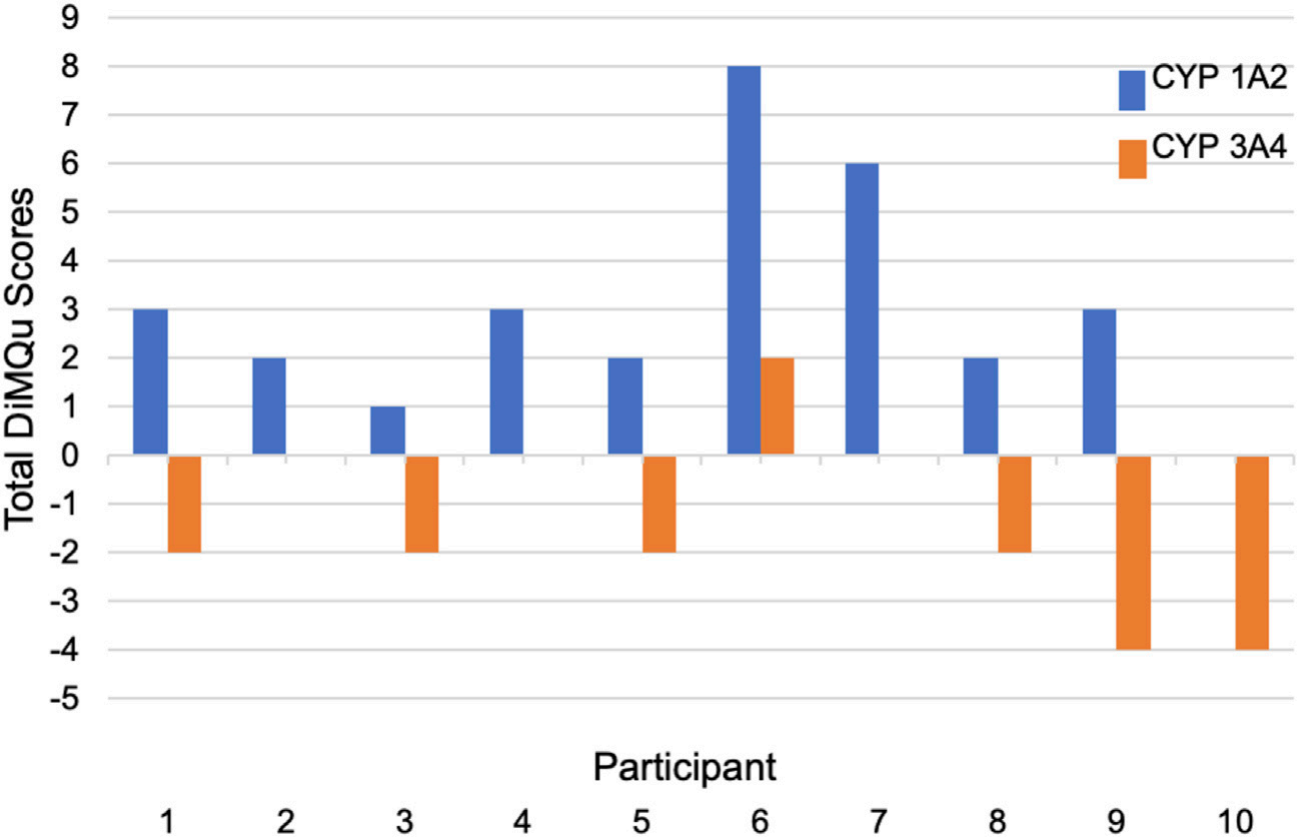
Calculated DiMQu scores for each participant.

Scores for DiMQu_3A4_ and DiMQu_1A2_ were calculated for each individual to provide a global prediction of the impact of their diet and medications on enzyme activity (Figure 1). No visible bar indicates a net score of zero. Positive scores were considered to be net inducing, whereas a negative score predicted inhibition. DiMQu_3A4_ scores predicted either no effect or a modest net inhibiting effect of diet and medication use on CYP3A4 enzyme activity for the majority of participants, whereas DiMQu_1A2_ scores predicted a net inducing effect principally driven by caffeine-containing products.

### 3.3 CYP P450 activity measurements

Details of the LC-MS/MS assay validation and associated quality controls are reported in Supplementary File S3. In brief, linearity, precision, and accuracy were assessed using charcoal-stripped serum spiked with increasing concentrations of caffeine/paraxanthine and midazolam/OH-midazolam reference standards. Peak area ratios for each analyte and its internal standard were fitted to a linear regression model with 1/x^2^ weighting. The assay underwent performance characterization and met acceptance criteria adapted from the United States Food and Drug Administration’s guidance for bioanalytical method validation (US-FDA, 2018). The linear range was 0.640–250 ng/mL for midazolam and OH-midazolam (R^2^ = 0.998), 20–5,000 ng/mL for caffeine (R^2^ = 0.999), and 10–2,500 ng/mL for paraxanthine (R^2^ = 0.999). Carryover contributed less than 20% of signal at the lower limit of quantitation (LLOQ) following injection of high calibrators, and no interferences were detected in matrix double blanks. Precision met criteria in that coefficients of variation (CVs) did not exceed 15% (or 20% at the LLOQ) for calibrators and quality control (QC) samples measured in triplicate. Recovery of spiked standards measured in three replicates at three concentration levels per analyte was on average 95%–99% for all analytes.

Each participant’s CYP3A4 Metabolic Ratio is presented in Table 3. As expected, all patient serum had midazolam and OH-midazolam levels below the lower limit of quantitation (LLOQ) at baseline. At 1-h, the mean concentration of free OH-midazolam was 6.26 ± 1.91 ng/mL whereas the mean concentration measured in the β-glucuronidase-treated samples was 54.0 ± 14.2 ng/mL, representing a difference of more than 8-fold. The MR for CYP3A4 varied from 5.09 to 13.4 or a 2.6-fold range from lowest to highest.

The CYP1A2 Metabolic Ratio for each participant is presented in Table 4. Despite instructions to fast completely prior to sampling, high concentrations of caffeine (up to 3,761 ng/mL) and paraxanthine (up to 3,297 ng/mL) are observed in some participants’ baseline serum samples. The MR for CYP1A2 varied from 0.04 to 0.14 or a 3.5-fold range.

**TABLE 4:** CYP1A2 Metabolic Ratio. The metabolic ratio is calculated as the change (∆) in paraxanthine at 1 h divided by the change (∆) in caffeine at 1 h.

		Caffeine (ng/mL)			Paraxanthine (ng/mL)			Metabolic ratio
				∆			∆	
		0 h	1 h	1 h	0 h	1 h	1 h	1 h
Participant								
1		316.7	2,246	1930	273.7	446.6	172.9	0.09
2		487.3	2,888	2,400	732.0	971.8	239.8	0.10
3		3,761	6,263	2,502	3,297	3,227	229.3	0.09
4		1747	5,705	3,957	1,528	1809	271.7	0.07
5		336.6	2,752	2,416	463.1	674.5	211.3	0.09
6		212.6	2,656	2,444	341.2	576.7	235.5	0.10
7		352.6	3,704	3,352	633.9	996.6	362.7	0.11
8		111.6	2,882	2,770	81.01	246.4	165.4	0.06
9		201.1	3,346	3,145	318.1	750.3	432.3	0.14
10		898.4	3,547	2,649	1,294	1,393	99.30	0.04

### 3.4 Correlation of predicted activation score to measured CYP activity

**FIGURE 2:**
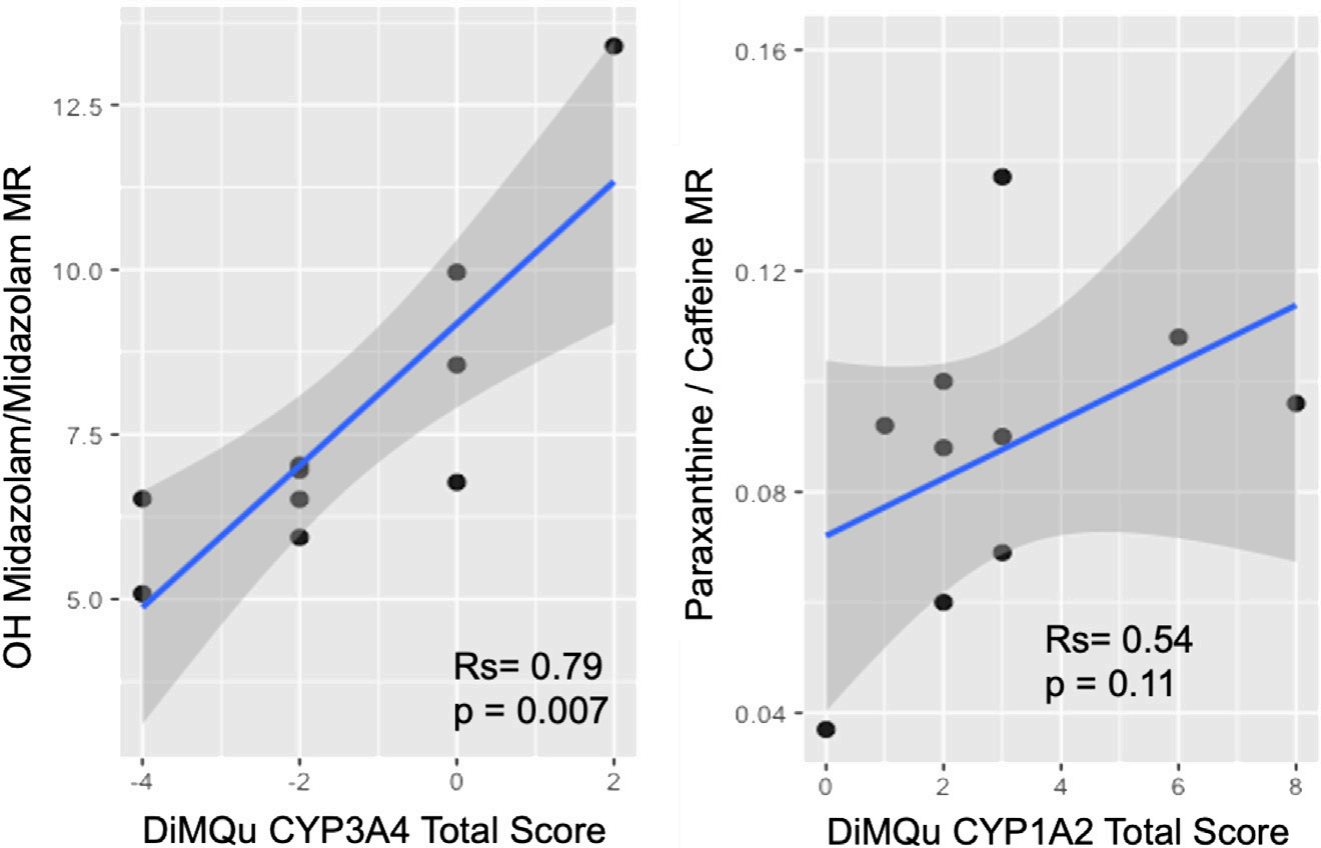
Correlation between DiMQu scores and metabolic ratio for CYP3A4 (left) and CYP1A2 (right).

The DiMQu score for CYP3A4 was strongly correlated (Rs = 0.79, *p* < 0.01) to the metabolic ratio of OH-midazolam:midazolam (Figure 2), but only in the β-glucuronidase-treated samples. However, the DiMQu score for CYP1A2 did not correlate to the paraxanthine:caffeine metabolic ratio (Rs = 0.54, *p* = 0.11, Figure 2). Participant age and participant sex were not significantly associated with the metabolic ratio phenotype for CYP3A4 or CYP1A2 (Supplementary Figures S4.1, 4.2). Metabolic ratios for CYP1A2 and CYP3A4 were not correlated with one another (Supplementary Figure S4.3).

### 3.5 Assay adaptation to dried blood spots

The linear range for the assay adapted to DBS was compatible with the range observed in patient samples (Table 5). On-spot deconjugation performed as well as aqueous extraction followed by β-glucuronidase treatment, yielding similar fold increases in OH-midazolam. The observed increase in OH-midazolam was 5.2-fold (on-spot) and 6.5-fold (aqueous extraction) for Whatman 903 cards, and 6.89-fold (on-spot) and 7.83-fold (aqueous) for HemaSpot HF (Supplementary Figure S5.1). All analytes were successfully quantified from as little as 18.4 µL of human blood, equivalent to two petals from a HemaSpot HF DBS collection device. This indicates the suitability of DBS for proxy measurements of CYP activity with the chemical probes used, as shown by others (Bosilkovska et al., 2014a).

**TABLE 5:** Validated assay performance for the analytical workflows applied to participant serum vs dried blood spots (DBS) prepared from whole blood.

Analyte	Observed serum concentration among participants (ng/mL)	Validated linear range in serum (ng/mL)	Validated linear range in pooled from whole blood extracted from DBS (ng/mL)
Caffeine	110–6,300^a^	20–5,000	80–4,000
Paraxanthine	80–3,300^a^	10–2,500	40–2000
Midazolam	4-12	0.1–24.4	0.4–20
OH-Midazolam	2-10	0.1–24.4	0.4–20

^a^ Where required, dilution was applied to permit quantitation of high concentration participant samples.

## 4 Discussion

The current study is the first to apply a modified version of the Geneva cocktail approach to healthy volunteers, and to link their free-living dietary and medication intake to the measured metabolic CYP phenotype. While previous Geneva cocktail studies induced enzymatic changes with the administration of inhibitors and inducers (Bosilkovska et al., 2014b), few studies using similar approaches have shown the effects of “everyday” nutritional factors (Le Marchand et al., 1997). Our findings highlight that common dietary exposures at “ordinary” levels of consumption can measurably impact drug metabolism. This clearly demonstrates the need for regular phenotypic assessment to capture changes in CYP activity due to exposure to modifiable external factors such as diet and medications that would not be captured using only pharmacogenetics screening. While a therapeutic drug monitoring (TDM) paradigm could also be used for phenotypic monitoring of cancer drug metabolism, the Geneva cocktail approach has the potential to permit dose-titration based on “real-time” assessment of CYP activity, *prior* to administration of a potentially toxic or ineffective dose of chemotherapy.

Our findings also inform application of the Geneva cocktail method. For instance, OH-midazolam is known to be metabolized to a OH-midazolam-glucuronide intermediate, but the extent to which this would occur in our study cohort was unknown. In comparing matched patient serum samples with and without β-glucuronidase treatment, we established that the majority of OH-midazolam (>85%) is in fact glucuronide-bound at 1 h after midazolam dosing. The correlation between the determined CYP3A4-activity and DiMQu score was observed only when considering the ratio of total OH-midazolam:midazolam after glucuronidase-treatment (*r*_*s*_ = 0.79, *p* = 0.007) and not for the ratio of free-OH-midazolam-to-midazolam (*r*_*s*_ = 0.06) alone. This result suggests that including the glucuronidase deconjugation step provides a more accurate measurement of actual CYP3A4-activity, despite the fact that this was not included in many prior studies applying the Geneva cocktail (Bosilkovska et al., 2014a; Bosilkovska et al., 2014b). This may also explain why other authors did not see correlation between apparent rates of midazolam conversion to 1-OH midazolam (measured without the glucuronidase treatment step) and clearance of erlotinib in patients with lung cancer, despite the fact that this drug is known to be extensively metabolized by CYP3A4 (Parra-Guillen et al., 2017). As shown in this study, the use of low volume DBS samples is a feasible option even with the method modifications proposed. This refinement is an important step making repeated sampling and longitudinal studies more practical. Future work should implement DBS sampling together with β-glucuronidase treatment to measure CYP activity in a broader cohort of cancer patients.

Generalization of the study results is limited by the small number of participants, which is likely not representative of the general population. Participants were mostly staff or graduate students at the Jewish General Hospital and none exceeded the age of 60. All were non-smokers and had BMIs <26, so expected relationships such as smoking inducing CYP1A2 activity (Anderson and Chan, 2016; Parra-Guillen et al., 2017) or obesity inhibiting CYP activity (Zarezadeh et al., 2021) could not be assessed. While the relationship between CYP activity and sex did not reach statistical significance, it is possible that there is a trend toward greater variability in the female participants; this could be due to induction/inhibition associated with greater medication use in females in our cohort, which reflects a wider disparity in prescription medication use (Desirée et al., 2013). No ethnicity or genetic data was obtained.

The developed assay demonstrates adequate performance for the clinical study of CYP-activity based on drug and metabolite ratios, though it is noted that there was no correlation between the metabolic ratio of paraxanthine to caffeine and the DiMQu_1A2_ score. Whilst a type II error cannot be excluded due to the small sample size, it seems likely that this may be due to interference in the measured metabolic ratio for CYP1A2 by high baseline caffeine and paraxanthine scores in some participants. Since only one timepoint at 1 h was analyzed to calculate the metabolic ratio, it is unclear whether the measured 1 h level was in fact an under-estimate of the true “peak,” particularly for individuals with high baseline levels. Though peak levels of caffeine occur around 1-h post ingestion, some data suggests later time points (>1 h) may be a better reflection of caffeine clearance (Derungs et al., 2016). However, it is also likely that the minimum 8-h avoidance of caffeine in the current study protocol is insufficient given the slower clearance of previously consumed caffeine and its metabolite paraxanthine (Samer et al., 2013; Bosilkovska et al., 2014a) and a minimum 24 h abstinence from caffeinated products prior to the final 8 h fast would have yielded more informative results. Known associations of CYP1A2 activity with age and sex were also not observed in this cohort, though some sources suggest that these effects may be marginal (Gunes et al., 2009). It is possible that the DiMQu_1A2_ score was inaccurate due to limits in the comprehensiveness of the dietary and medication questionnaire, incomplete or inaccurate reporting, and failure to avoid caffeinated fluids the morning of the test (as suggested by the very high caffeine levels in some participants). It is also worth mentioning that metabolism of caffeine and its secondary metabolites are regulated by polymorphisms of CYP1A2 and other critical enzymes, which have been the subject of previous genotyping studies (Nehlig, 2018). While the details of caffeine’s metabolism are outside of the scope of this study, its complexity may contribute to the observed results. Given the challenges noted above, consideration could also be given to using a different CYP1A2 substrate such as phenacetin (Spaggiari et al., 2014).

There is clear evidence for the impact of diverse influences on CYP activity, but extensive research will be required to develop accurate models that meaningfully incorporate genetic and non-genetic factors, and accurately predict their effects. (Daali et al., 2021). The interplay between contributors to CYP activity is notoriously complex (Storelli et al., 2018). In the meantime, we have implemented a streamlined version of the Geneva cocktail approach for direct real-time monitoring of CYP activation that is simple, cost-effective, safe, and avoids the need for genetic testing. The DiMQu scoring scale may help flag patients for whom phenotyping is required. The phenotyping method used was also successfully translated to DBS for ease of implementation in a busy hospital setting and use in large-scale clinical studies. Transfer of the reported assay to DBS will facilitate sample-collection and storage, while minimizing patient discomfort. While the main goal of this study is to lay the groundwork to enable routine monitoring, the newly emerging role of CYPs in oncogenesis also suggests a broader potential application of CYP activity characterization in precision medicine to not only dose, but to actually help select appropriate targeted treatments (Rodriguez-Antona et al., 2010).

## 5 Conclusion

This study reports one of the first uses of a simplified Geneva cocktail approach to phenotype the activity of CYP1A2 and CYP3A4 in healthy free-living male and female study volunteers. Our results demonstrate that glucuronide deconjugation is crucial for CYP3A4 phenotyping with this approach. The DiMQu induction score created to predict enzyme activation from a dietary questionnaire appears to effectively integrate information about inducers and inhibitors, and also correlated with observed metabolic phenotype for CYP3A4. The results of the CYP3A4 phenotyping and the DiMQu scores reinforce the wide variation in CYP3A4 activity that is likely caused by external factors such as diet and medication even in free-living participants. It seems likely that there would be even greater variation in CYP activity in patients undergoing cancer treatments and there would be considerable clinical value in being able to measure this when making decisions about drug dosing and predictions of toxicity and efficacy. Our protocol for DBS analysis represents the first reported method for on-spot deconjugation of glucuronide metabolites in the context of CYP activity monitoring. The DBS method will help facilitate more routine implementation in the clinic to ultimately support dose optimization for better cancer therapies.

## Data Availability

The original contributions presented in the study are included in the article/Supplementary Material, further inquiries can be directed to the corresponding author.
