# Supplementary material for: Measurement of CYP1A2 and CYP3A4 activity by a simplified Geneva cocktail approach in a cohort of free-living individuals: a pilot study
Source: Front Pharmacol. 2024 Feb 2;15:1232595. doi: 10.3389/fphar.2024.1232595 (PMC10869543; doi:10.3389/fphar.2024.1232595)
Supplement: Supplementary file 1 [file Presentation1.pdf]

Supplementary Material

1 Dietary & Medication Questionnaire

QUESTIONNAIRE

		Insert check mark in the boxes which best represent your intake					
**Food/Drink**	**Serving Size**	**Never or Rarely**	**1-3 per month**	**1-3 per week**	**4-6 per week**	**1-2 times a day**	**3 or more times a day**
Coffee, regular	1 cup (250 mL)						
Coffee, decaffeinated	1 cup (250 mL)						
Tea, regular	1 cup (250 mL)						
Tea, decaffeinated	1 cup (250 mL)						
Carbonated Soda: e.g. Coke, Pepsi, 7UP, Sprite, Root Beer, Dr. Pepper	1 can (355 mL)						
Cruciferous Vegetables: e.g. Cauliflower, Broccoli, Cabbage, Bok Choy, Brussel Sprouts, Turnips, Radish	1 cup of vegetables						
Charbroiled, barbequed, or pan-fried meat: e.g. Chicken, Beef, Pork, Sausage, Lamb, Turkey	1 portion (3oz = about size of your palm)						
Red Wine	1 glass (5oz/ 150ml)						
Beer	1 bottle/can(12oz /355 ml)						
Spirits/Liquor	1 shot (1.5 oz/ 44ml)						
Garlic cloves	1 serving						
Grapefruit / Seville oranges	1 medium-sized orange						
Jufeng Grape Juice	1 cup (250 mL)						
Liquorice Candy	1 medium-sized pack of candy						
Indian food	1 serving						
Ground black pepper (for seasoning/taste in a meal)	-						

Smoking

Do you smoke (please circle one)? (Y / N)

How many cigarettes do you smoke in a day?

____

Medical History:
Please list any current chronic medical problems or conditions that you have. If none, please write 'none.'

Complementary Natural Health Products:
Please list any complementary or natural health products that you take (example: curcumin, herbal products, vitamins, supplements, spices, etc.).

Name of Product	Amount of Product Taken	How Often Is It Taken

Current Medications:
Please list any medications you are currently taking, whether prescription or over the counter (Please remember to include any hormonal treatments, birth control, etc.).

Name of Medication	Dose of Medication	How Often Is It Taken?	How Do You Take it?

Time of first blood test: ____________________

Time of second blood test: ____________________

Height: ____________________

Weight: ____________________

2 Categorical Assignment of Inhibitors / Inducers

Table 1. Categorical Assignment of Inhibitors / Inducers from Dietary Exposures

	CYP1A2		CYP3A4	
Item	Inducer	Inhibitor	Inducer	Inhibitor
Coffee	✓			
Tea	✓			
Red Wine*		✓		✓
Beer*		✓		✓
Spirits/Liquor*		✓		✓
Carbonated Soda	✓			
Grapefruit / Seville oranges				✓
Cruciferous Vegetables	✓			
Charbroiled, barbequed, or pan-fried meat	✓			
Jufeng Grape Juice	✓			
Indian food (curcuma)*		✓		✓
Liquorice Candy				
Ground black pepper				✓
Garlic			✓	

Note. Coffee and tea includes decaffeinated options. Meats include: chicken, beef, pork, sausage, lamb and turkey. Cruciferous vegetables include: cauliflower, broccoli, cabbage, bok choy, brussel sprouts, turnips, radish. * indicates minor inhibitor or inducer

Minor(*) inhibitors or inducers were assigned a strength score of 1. For all others, strength = 2.

Table 2. Categorical Assignment of Inhibitors / Inducers from Patient-Report Medications & Supplements

	CYP1A2		CYP3A4	
	Inducer	Inhibitor	Inducer	Inhibitor
Modafinil	✓		✓	
Curcumin*		✓		✓
Omega 3 Fatty Acid		✓		
Melatonin		✓		
Loratidine			✓	
Oregano Oil				✓
Teva-venlafaxine XR				✓

*Note.** indicates minor inhibitor or inducer

Minor inhibitors or inducers were assigned a strength score of 1. For all others, strength = 2.

3 LC-MS/MS Assay Details

The same LC-MS method was applied for both method validation and sample analysis, with details as shown below:

MS Transitions & Collision Energies

Analyte	Transition	CE
Hydroxymidazolam (IS)	346/328	22
Hydroxymidazolam (IS)	346/203	31
Hydroxymidazolam	342/324	22
Hydroxymidazolam	342/203	31
Midazolam (IS)	330/295	28
Midazolam (IS)	330/253	40
Midazolam	326/291	28
Midazolam	326/249	40
Caffeine (IS)	204/144	21
Caffeine (IS)	204/116	27
Caffeine	195/138	21
Caffeine	195/110	27
Paraxanthine (IS)	187/127	20
Paraxanthine	181/124	20

MS Settings

Parameter	Settings
System	Agilent 6495 MS/MS
Ion Source	AJS ESI
Method	MRM-MS
Delta EMV (+)	400
Source gas temp	150 C
Gas Flow	15 l/min
Nebulizer	30 psi
Sheath Gas Temp	250 C
Sheath Gas Flow	11 l/min
Polarity	positive
Capillary	3500V + / 3000 V neg
Nozzle voltage	300 V pos / 1500 V neg
Resolution (MS1 and MS2)	Unit Res
Fragmentor	380
Cell Accelerator Voltage	5
Cycle time	3.04 cycles/s, 329 ms/cycle (dwell 20/transition)

Time filtering	Peak width 0.03 min

Sample chromatograms

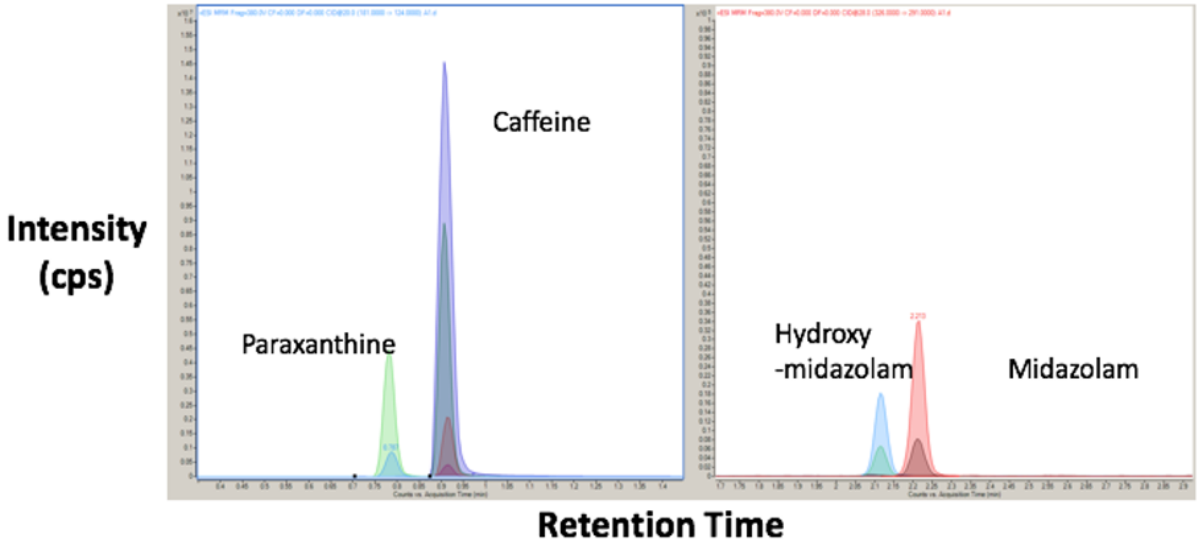

Linearity, precision, and accuracy were assessed using charcoal-stripped serum spiked with increasing concentrations of caffeine/paraxanthine and midazolam/OH-midazolam reference standards. The peak area ratios were fitted to a linear regression model with $1/x^2$ weighting. Assay linearity was established from 640 pg/mL-250 ng/mL for midazolam and OH-midazolam (R^2=0.998), 20 ng/mL-5 µg/mL for caffeine (R^2=0.999), and 10 ng/mL-2.5 µg/mL for paraxanthine (R^2=0.999). Carryover was below 20% of the LLOQ following injection of high calibrators, no interferences were detected in matrix double blanks. Precision for all calibrators and QC samples were within 15% and 20% CV at the LLOQ.

Analyte	Desired Range (ng/mL)	Achieved Linear Range (ng/mL)	Number of points in curve	Number of QC passed	Linearity (R^2)	CV at LLOQ (%)
Caffeine	25-5000	20-5000	7	3/3	0.9914	<15
Paraxanthine	25-5000	10-2500	7	3/3	0.9942	<15
Midazolam	0.1-100	0.10-24.4	6	3/3	0.9990	<15
Hydroxymidazolam	0.2-200	0.10-24.4	6	3/3	0.9950	<15

Note. Average coefficient of variation (CV) is of 3 replicates prepared in parallel at each level included in linear range. Quality Control (QC) is set at high, medium and low.

OH-midazolam is known to be metabolized to OH-midazolam-glucuronide, but the extent to which this would occur in our study cohort was unknown. To address this, we split each patient sample into a β-glucuronidase-treated and control group. The mean concentration of free vs glucuronide

conjugated OH-midazolam was found to be 0.7 and 4.8 ng/mL respectively representing a 7-fold ratio difference.

The performance characteristics of the LCMS assay from serum are summarized in the table above and confirm that the method works well and can be used for clinical studies. The assay linear range that was sufficient for the expected range of analyte concentrations. The coefficient of variation (CV) for repeated testing was checked at lower limit of detection and was <15% for all analytes which meets the FDA standard (LLOQ <20%). The coefficient of variation at the lower limit of quantification (LLOQ) determines the assay's precision. The quality control points (low/medium/high) all fell within the criteria (known concentration ± 15%). These quality control points are samples with known concentrations that are independent of the standard curve. The use of charcoal stripped serum allowed a blank sample containing no caffeine and paraxanthine (which are commonly seen in blood samples, even criteria. Each standard curve included at least 6 points within the linear range and demonstrated high linearity (R2>0.985). The signal for each analyte in the blank charcoal-stripped serum was less than 20% of the signal at the lower limit of quantification. Also, the response for both analytes and internal standards in the double blank solvent was lower than 20% of the signal at the lower limit of quantification, showing that no carryover occurred during analysis. The transition ratios were consistent between labeled and unlabeled standards, showing that the samples were consistent with calibrators and quality controls and did not contain unexpected interferences.

<u>Accuracy.</u> With respect to the accuracy of concentration measurements, the measured concentration of calibration standards based on the line of best fit, was accurate within +/-20% for each standard within the linear range for each analyte. Quality control samples (high/medium/low), consisting of charcoal-stripped serum spiked with known quantities of each analyte, were included with each batch and the measured concentration in these samples was also accurate within +/-20% of the known value.

<u>Recovery</u>. The recovery for each analyte was assessed by comparing the quantitation of standards in buffer, spiked into previously-extracted charcoal-stripped serum, and spiked into charcoal-stripped serum before extraction. The recovery of standards spiked prior to extraction was measured in 3 replicates at 3 concentration levels per analyte, as shown below.

**Analyte**	**Level (fmol on-column) & Recovery**					
Sample	High		Medium		Low	
**Paraxanthine**	**Std B**	**2000**	**Std D**	**320**	**Std F**	**51.2**
Buffer	102%		97%		97%	
Spiked into extracted	121%		104%		107%	
Spiked & recovered	102%	±2%	94%	±2%	91%	±3%
**Caffeine**	**Std B**	**1000**	**Std D**	**160**	**Std F**	**25.6**
Buffer	97%		101%		98%	
Spiked into extracted	117%		103%		102%	
Spiked & recovered	104%	±3%	91%	±1%	91%	±1%
**OH-midazolam**	**Std B**	**10**	**Std D**	**1.6**	**Std F**	**0.26**
Buffer	101%		95%		98%	
Spiked into extracted	115%		93%		121%	
Spiked & recovered	97%	±6%	95%	±12%	104%	±2%

Midazolam	Std B	10	Std D	1.6	Std F	0.26
Buffer	103%		100%		100%	
Spiked into extracted	115%		91%		98%	
Spiked & recovered	103%	±2%	84%	±1%	97%	±6%

Reproducibility. The reproducibility of the assay was further demonstrated since each of the 3 sets of technical replicates was analyzed during the assay validation process. For each analyte, known concentrations of the standard were added into the serum at 3 levels (low, medium, high). For each level, three identical samples were extracted and analyzed in parallel. The resulting reproducibility testing at different concentrations within the linear range of the assay showed CV was <7%. These performance indicators ensure the validity of the measured concentrations for this study.

Stability. Due to the necessity of sample storage, the analytes' stability over time needed to be assessed. Samples analyzed over 90 days of storage at -80ºC showed agreement in the values generated from analysis (CVs <6%) confirming 3-month stability.

4 Metabolic Ratio versus Participant Age and Sex

Figure 4.1. Scatterplots for MR vs Age for CYP3A4 (left) and CYP1A2 (right)

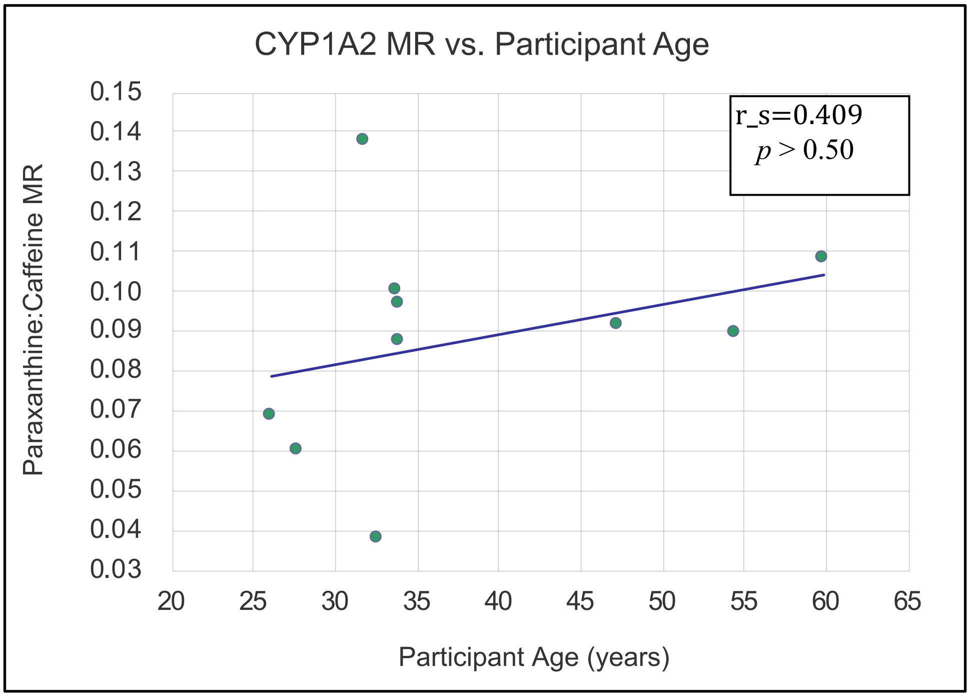

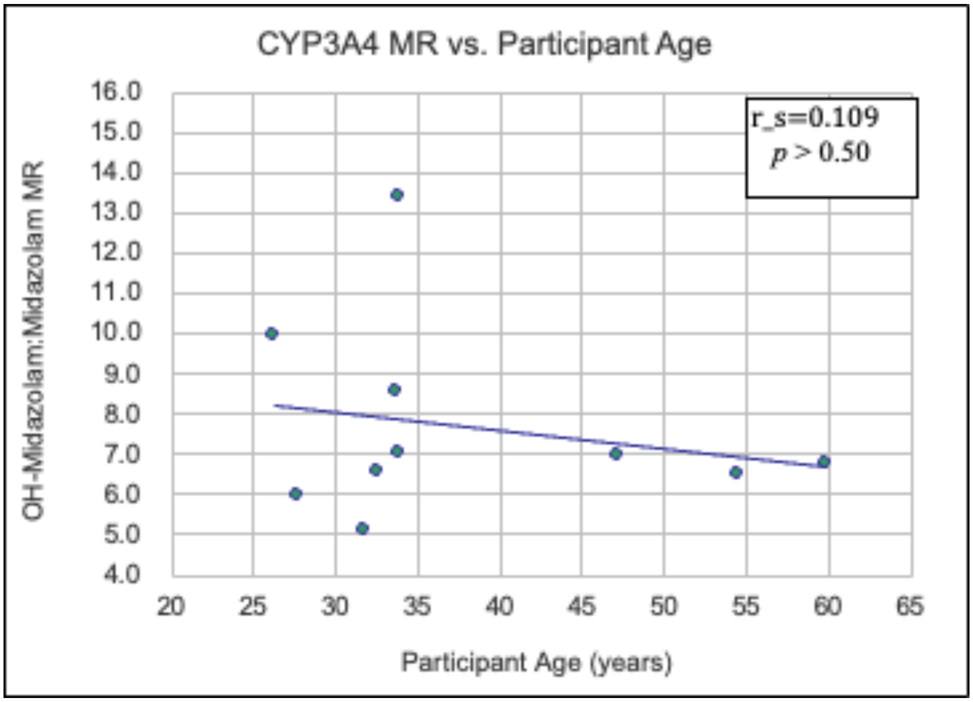

Figure 4.2. Metabolic ratio of probe versus sex for CYP3A4 (left) and CYP1A2 (right)

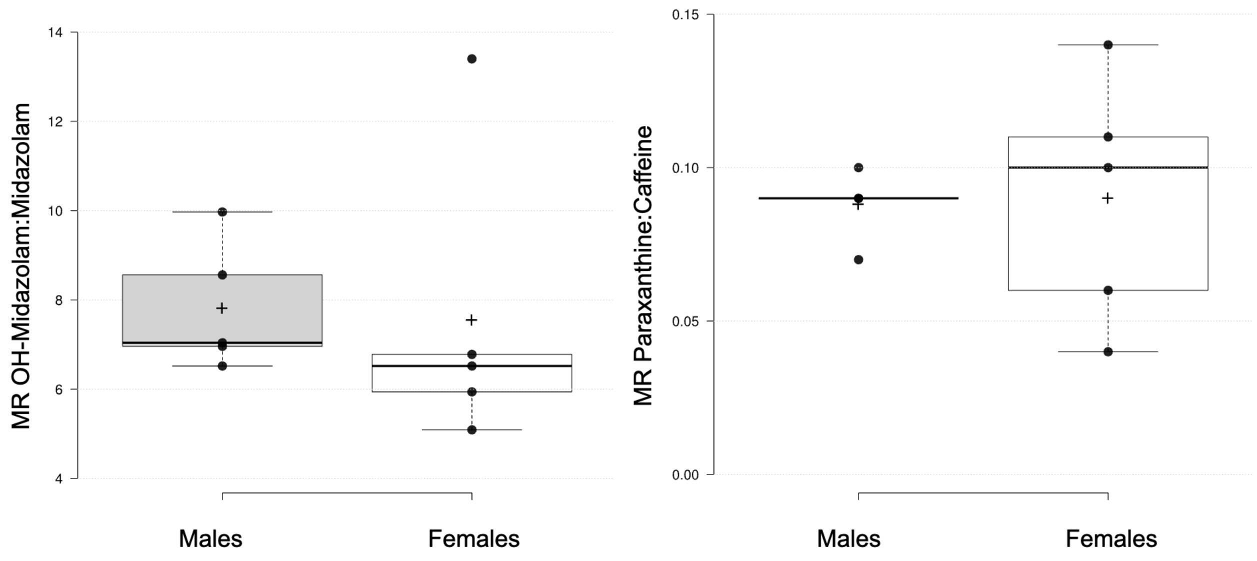

Figure 4.3 CYP1A2 MR vs. CYP3A4 MR

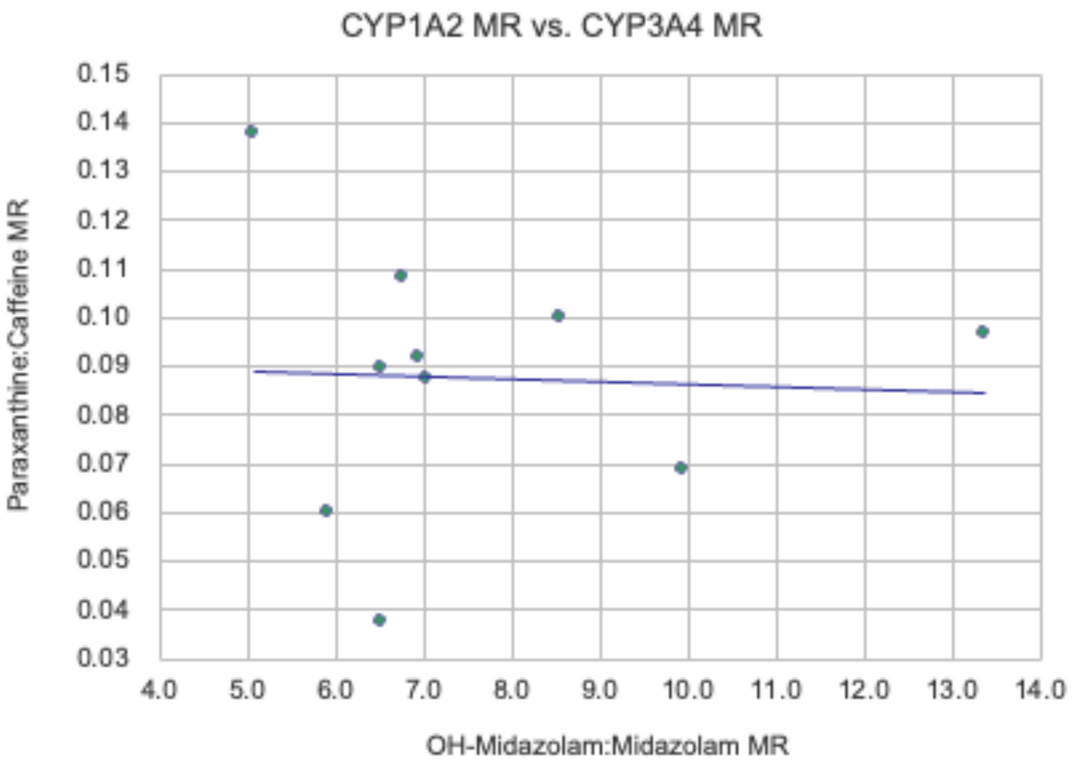

5 Assay Performance in DBS

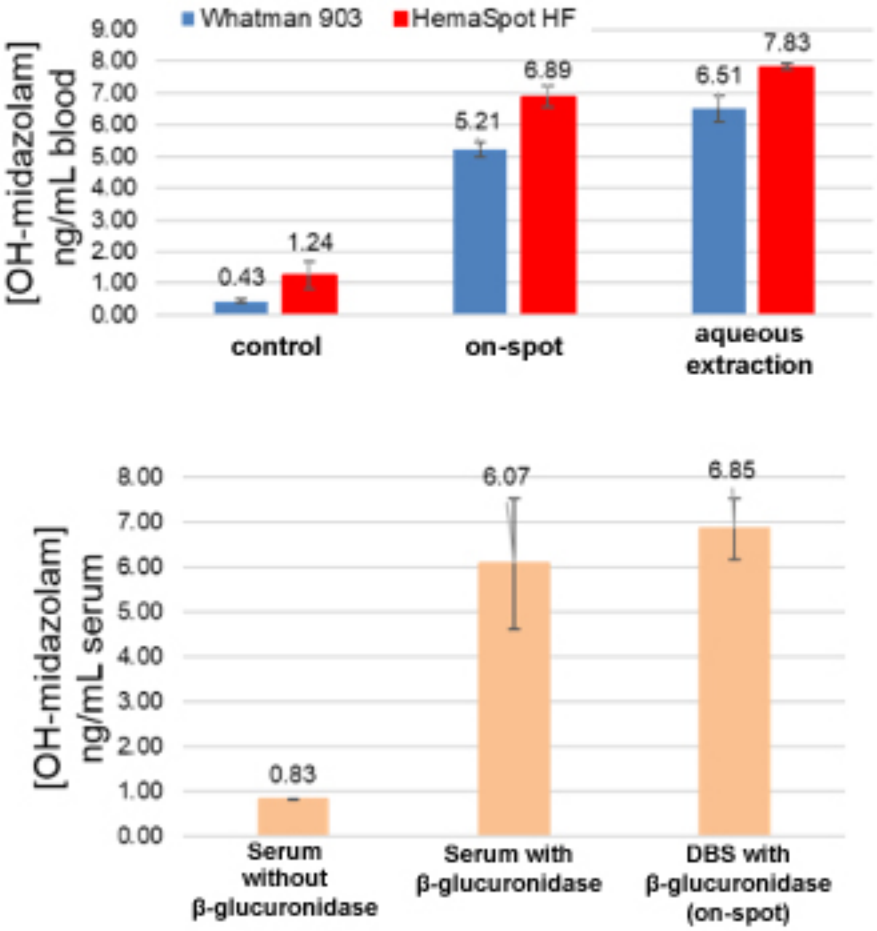

Figure 5.1 Comparison of direct ("on-spot") and sequential ("aqueous extraction") extraction and deconjugation of OH-midazolam glucuronide. Top panel: Dried blood spot (DBS) samples spotted on either Whatman 903 (blue bars) or HemaSpot HF (red bars) substrates were either: (i) incubated 16 hours at 37°C without β-glucuronidase ("control"), (ii) incubated with 500 units of β-glucuronidase for 16 hours at 37°C ("on-spot"), or (iii) extracted from the DBS aqueous extraction followed by deconjugation ("aqueous extraction"). Bottom panel: Measurement of OH-midazolam from pooled serum controls vs. dried blood spot samples. Serum samples (50 µL) were either (i) incubated 16 hours at 37°C without β-glucuronidase, or (ii) incubated with 500 units of β-glucuronidase for 16h at 37°C, vs. (iii) DBS generated from pooled whole blood that were extracted and treated with β-glucuronidase using the "on-spot" method.
